# Study protocol: Effectiveness of dual-mobility cups compared with uni-polar cups for preventing dislocation after primary total hip arthroplasty in elderly patients — design of a randomized controlled trial nested in the Dutch Arthroplasty Registry

**DOI:** 10.1080/17453674.2020.1798658

**Published:** 2020-08-04

**Authors:** Loes W A H Van Beers, Bart C H Van Der Wal, Tess Glastra Van Loon, Dirk Jan F Moojen, Marieke F Van Wier, Amanda D Klaassen, Nienke W Willigenburg, Rudolf W Poolman

**Affiliations:** a OLVG, Amsterdam;; b University Medical Center Utrecht, Utrecht;; c LUMC, Leiden, the Netherlands

## Abstract

Background and purpose — Dislocation is the leading reason for early revision surgery after total hip arthroplasty (THA). The dual-mobility (DM) cup was developed to provide more stability and mechanically reduce the risk of dislocation. Despite the increased use of DM cups, high-quality evidence of their (cost-)effectiveness is lacking. The primary objective of this randomized controlled trial (RCT) is to investigate whether there is a difference in the number of hip dislocations following primary THA, using the posterolateral approach, with a DM cup compared with a unipolar (UP) cup in elderly patients 1 year after surgery. Secondary outcomes include the number of revision surgeries, patient-reported outcome measures (PROMs), and cost-effectiveness.

Methods and analysis — This is a prospective multicenter nationwide, single-blinded RCT nested in the Dutch Arthroplasty Registry. Patients ≥ 70 years old, undergoing elective primary THA using the posterolateral approach, will be eligible. After written informed consent, 1,100 participants will be randomly allocated to the intervention or control group. The intervention group receives a THA with a DM cup and the control group a THA with a UP cup. PROMs are collected preoperatively, and 3 months, 1 and 2 years postoperatively. Primary outcome is the difference in number of dislocations between the UP and DM cup within 1 year, reported in the registry (revisions), or by the patients (closed or open reduction). Data will be analyzed using multilevel models as appropriate for each outcome (linear/logistic/survival). An economic evaluation will be performed from the healthcare and societal perspective, for dislocation and quality adjusted life years (QALYs).

Trial registration — This RCT is registered at www.clinicaltrials.gov with identification number NCT04031820.

Dislocation after total hip arthroplasty (THA) is the leading reason for early revision surgery (Bozic et al. 2009, Gwam et al. 2017). Most dislocations occur during the first year after surgery, of which approximately half within the first 3 months (Woo and Morrey 1982, Phillips et al. 2003, Meek et al. 2006, Hailer et al. 2012). Especially in patients with recurrent dislocation and the need for revision surgery, this leads to reduced physical functioning and quality of life (Enocson et al. 2009). Dislocations also increase healthcare costs (Sanchez-Sotelo et al. 2006, Abdel et al. 2015). A single dislocation adds 19% to the hospital costs of an uncomplicated THA, and of a revision surgery up to 148% (Sanchez-Sotelo et al. 2006).

Despite the increased and, in some countries, broad use of DM cups, high-quality evidence of their effectiveness is lacking (Darrith et al. 2018). Recent reviews did not identify any randomized controlled trials (RCT) comparing DM cups with UP cups (De Martino et al. 2017a, 2017b, Darrith et al. 2018, Jonker et al. 2020) and the existing studies are of low methodological quality and at high risk of bias due to the lack of experimental design. So far only one—non randomized—cost-effectiveness study has been performed, suggesting that the DM cup may result in cost savings compared with a UP cup (Epinette et al. 2016). Although promising, the results of this cost-effectiveness database study are not transferrable outside France.

Therefore we initiated an RCT to establish the effectiveness of DM cups for primary THA. The primary objective is to investigate whether there is a difference in the number of hip dislocations following primary total hip arthroplasty (THA), using the posterolateral approach, for a DM cup compared with a UP cup in elderly patients within 1 year after surgery. Several secondary outcomes will be specified in the methods section. The registry-nested design will facilitate long-term follow-up for all study participants.

## ^Methods and analysis^

### ^Study design^

This is a prospective registry-nested multicenter single-blinded RCT, which will be conducted in 10 general and academic hospitals in the Netherlands. This RCT compares the number of hip dislocations following primary THA with a DM cup compared with a UP cup and is nested in the Dutch Arthroplasty Registry (LROI).

All patients will be followed up until 2 years after surgery. The recruitment phase started in April 2019 and was anticipated to last 2.5 years. After the first year of recruitment, we have experienced a slight delay. After final study follow-up, participants remain traceable in the LROI for evaluation of long-term survival and mortality.

### ^Participants^

All patients at the orthopedic outpatient clinics of participating centers that meet the criteria to undergo an elective primary THA will be screened for the in- and exclusion criteria.

Patients can be included when they are 70 years or older; have adequate comprehension of written and spoken Dutch; and are eligible for elective primary THA with a cup large enough for a 32 or 36 millimeter head diameter, by a surgeon who is comfortable using the posterolateral approach. A previous contralateral THA is not a reason for exclusion, but patients who undergo bilateral hip arthroplasty can only participate in the trial with 1 of the hips. Patients will be excluded when they: are not able to complete PROMs; are not eligible for either a UP or DM cup; have epilepsy, spasticity, dementia, mental retardation, or alcoholism. If dementia or mental retardation is not already mentioned in the medical chart, this can be determined by doctor’s opinion.

Characteristics that will be collected are: age; sex; BMI; smoking; diagnosis; ASA classification; Charnley score; education level according to the Statistics Netherlands classification; surgical details (e.g., side, any complications); implant details (e.g., brand, size); type of fixation (cemented or uncemented); type of stem.

### ^Interventions^

All patients participating in the RCT will be treated with a THA using the posterolateral approach. Patients are randomly allocated to a DM cup or to a UP cup with a 1:1 allocation ratio. It is a requirement for participating surgeons to feel confident with both procedures. The Dutch guidelines recommend reconstruction of the capsule and external rotators when using the posterolateral approach. There are no restrictions to a specific brand of implant, and participating hospitals can use the implants of the companies they usually work with. This study does not investigate any specific implant, but rather pragmatically the concept of DM cups. The Avantage (Zimmer Biomet, Warsaw, IN, USA) and POLAR (Smith & Nephew, London, UK) cups are examples of commonly used DM cups. The IP (Link, Hamburg, Germany), FAL (Link), Exeter (Stryker, Kalamazoo, MI, USA) and Pinnacle (Johnson & Johnson, New Brunswick, NJ, USA) cups are commonly used UP cups. Cemented DM and UP cups have 5-year survival rates of ≥ 96%, with cumulative revision rates ranging from 1.9% to 4.0% when revision was defined as any change (insertion, replacement, and/or removal) of one or more components of the prosthesis, for any reason (LROI 2017b). Lubinus SP2 (Link), Exeter (Stryker), and Corail (Johnson & Johnson) are the commonly used stems.

All patients receive the same standard pre- and postoperative care for both DM and UP cups according to their hospital’s standard.

### ^Sample size calculation^

Exact dislocation rates in the Netherlands are unknown, as only those dislocations that result in revision surgery are registered. Based on previous studies and reviews, we assume that the current dislocation rate for UP cups is 4% whereas DM cups result in 1% dislocation (Philippot et al. 2009b, Boyer et al. 2012, Fresard et al. 2013, Prudhon et al. 2013, Caton et al. 2014, Batailler et al. 2017, De Martino 2017a). Power analysis indicates that a total sample of 976 (488 in each group) is needed to detect a difference in dislocations between 4% in the UP cup group and 1% in the DM cup group, using the chi-square test with 80% power and α = 0.05. To account for loss to follow-up, 550 patients will be included in each group.

### ^Outcomes^

#### Primary outcome

The primary outcome is the number of hip dislocations, regardless of type of treatment. This information is collected from both the LROI and the patient. Since the LROI registers only revisions, open and closed reductions would be missed. Therefore, patients are asked with a questionnaire at 3 months, 1- and 2-year follow-up whether they have had a hip dislocation.

#### Secondary outcomes

Secondary outcomes are any unplanned hip procedures, including revision surgery of any component, for any reason; cost-effectiveness; and PROMs.

The following PROMs are collected preoperatively, and 3 months, 1 and 2 years postoperatively: Physical functioning of the hip measured with the Hip disability and Osteoarthritis Outcome Score Physical Short form (HOOS-PS) (de Groot et al. 2007); Quality of life measured with the EuroQol 5 Dimensions (EQ-5D) (EuroQol 1990); pain measured with a numeric rating scale (NRS) ranging from 0 to 10 for pain at rest and during weight-bearing; change in physical functioning measured with an anchor question; fear of hip dislocation measured on a five-point Likert scale. At all postoperative moments, the awareness of type of cup that was placed is asked.

At 3 months and 1 year postoperatively healthcare and societal costs related to hip dislocation or surgery are measured with a retrospective 4-week cost evaluation questionnaire, which is filled out by the patient. We will obtain information on health care utilization, (pain) medication used, patient costs, use of domiciliary care, use of informal care, and sickness absenteeism from paid or unpaid work. Healthcare utilization consists of general practitioner care, allied healthcare, medical specialist care, imaging tests, admission to a hospital, rehabilitation center, nursing home or care home, and mobility aids. Participants’ costs concern the patient contribution towards costs for mobility aids and travel. Domiciliary care consists of home nursing care and home help. Healthcare utilization, domiciliary care, informal care, and sickness absenteeism will be valued at Dutch standard costs (Hakkaart-van Roijen et al. 2015). If these are not available, prices reported by professional associations will be used. The costs of prescribed medications will be calculated using prices charged by the Royal Dutch Society for Pharmacy.

### ^Study procedures^

#### Informed consent

During the preoperative visit at the outpatient clinic, patients who are potential candidates for this study will be screened to determine whether they meet the in- and exclusion criteria. If the patient is eligible, the investigator (or his designated representative) will propose participation in the study to the patient, according to GCP guidelines. Patients must sign an informed consent form approved by the ethical committee, prior to participating in any study-specific related activities.

#### Randomization

After signing informed consent, 1,100 patients will be randomized to either the intervention group (DM cup) or the control group (UP cup). Each group will consist of 550 patients. The investigator (or his designated representative) will perform the randomization using the platform CASTOR Electronic Data Capture (www.castoredc.com). Variable randomization blocks of 2, 4, and 6 patients will be used, and we shall stratify for center. Patients will be blinded for treatment allocation. The participating surgeons may divert from the randomization scheme based on intraoperative findings. Any deviation from the assigned treatment group will be reported as a deviation from the protocol.

#### Follow-up

Patients are evaluated at 3 months, 1 year and 2 years after surgery.

### ^Data analysis plan^

#### Interim analysis

Interim analysis for the primary study outcome will be performed when 200 patients have reached the 3 months postoperative PROM evaluation point. In the interim analysis the number of dislocations in each group will be compared. A chi-square test will be used and in any case where the assumptions of this test are not met, Fisher’s exact test will be applied. To guard against a type 1 error, we will use the O’Brien–Fleming approach. As only 1 interim analysis will be performed, the alpha for this analysis is set at 0.005. Testing will be done 2-sided. Furthermore, we will consider the number of revisions and SAEs in each group, but not formally test for differences in these. Results of the interim analysis will be discussed with the study team, the Van Rens Foundation (funder of this study), and the ethical committee. In the case of a statically significant and relevant higher number of dislocations in the DM group, or more revisions or SAEs, appropriate actions will be taken (such as an early termination of the study).

#### Primary outcome analysis

The primary outcome, the difference in number of dislocations in both groups, will be analyzed using chi-square analysis. Additional exploratory multivariable logistic regression analyses will adjust for clustering of data (e.g., at the hospital level), and possible confounding or effect modification of patient and surgical characteristics (e.g., age; sex; BMI; smoking; diagnosis; ASA classification; Charnley score; education level according to the Statistics Netherlands classification; surgical details; implant details; type of fixation; type of stem). A multilevel survival model will be used to analyze the survival of the implant, corrected for covariates.

Analyses will be performed using both intention-to-treat as well as per-protocol analysis.

#### Missing values

Efforts will be made to prevent missing data by sending reminders and making phone calls when appropriate. A reasonable amount of dropouts is anticipated, and mixed-model analyses will account for missing data using maximum likelihood estimation. In the event of unforeseen numbers of missing values, a state-of-the-art solution will be sought in consultation with a statistician (e.g., imputation, depending on the nature of the missing data).

#### Secondary outcomes analyses

Secondary study outcomes are any surgical intervention on the affected hip including revision surgery, healthcare costs, societal costs, patient-reported physical functioning, quality of life, pain, satisfaction, fear of hip dislocation and device-related complications and reoperations. The secondary outcomes will be analyzed using similar multilevel models as appropriate for each outcome (linear/logistic/survival).

An economic evaluation will be performed from the healthcare and societal perspective, for dislocation and quality adjusted life years (QALYs). Prevailing guidelines of Zorginstituut Nederland will be observed. All costs and consequences relevant to THA, hip dislocation, and hip revision will be accounted for.

To compare costs between groups, confidence intervals around the mean differences in costs at one year after THA will be estimated using the bias-corrected and accelerated bootstrap method. To account for possible clustering of data and to adjust for possible confounders, multi-level analyses will be performed. To present the incremental cost-effectiveness ratios and uncertainty around them graphically, bootstrapped cost-effect pairs will be plotted on cost-effectiveness planes. Cost-effectiveness acceptability curves will present the probability that the DM cup is more cost-effective than the UP cup for a range of willingness-to-pay thresholds. To study the robustness of these results, sensitivity analyses will be performed.

## ^Discussion^

To the authors’ knowledge, this is the first RCT comparing UP and DM cups for primary THA. In contrast to the observational nature of all (registry) studies to date, this study will be able to draw causal inferences. Previous literature is mostly from France, where DM cups are already used in approximately 30% of all primary THAs (Epinette et al. 2016). Dislocation rates seem lower for dual mobility (DM) cups (range 0% to 3.6%) than for unipolar cups (range 0.5% to 6%) (van der Grinten and Verhaar 2003, Bourne and Mehin 2004, Jolles and Bogoch 2004, Malkani et al. 2010, Lachiewicz and Soileau 2013, Dargel et al. 2014). Good results are also shown when DM cups are used in revision surgery for patients with recurrent dislocation (Langlais et al. 2008, Philippot et al. 2009a, Hailer et al. 2012). The Dutch Arthroplasty Registry shows that 3.9% of all cemented cups in 2015 were DM cups (LROI 2017a). The proportion of DM cups in all primary THA increased from 0.8% in 2010 to 2.6% in 2016 (Bloemheuvel et al. 2019). In the Netherlands and other countries, DM cups are typically used for primary THA in patients with specific characteristics, such as cognitive impairment (not able to follow restrictions after surgery), neuromuscular diseases (spasms), or alcohol abuse, or as a standard procedure for revision surgeries due to recurrent dislocations (De Martino et al. 2017a, Bloemheuvel et al. 2019). These patient characteristics might negatively influence the risk for dislocation and revision surgery, so data of these specific patient groups cannot be generalized to the regular primary THA population.

Our registry-nested randomized design is an efficient way to obtain an unbiased comparison between DM and UP cups, both in the short term and long term. Currently, dislocations are only reported in the registry if they result in implant revision. Therefore, the primary—relevant to patients—outcome of this study is a composite measure of revisions due to dislocation reported in the registry and patient-reported dislocations that were treated with closed or open reduction. Not many studies used such a composite outcome, which complicated our sample size calculation. The current group sizes are based on informed assumptions, and considered large enough to detect substantial differences between groups. However, regarding this limitation we believe it is fair to compare groups in terms of dislocation rates with corresponding confidence intervals rather than strictly focusing on p-values (Wasserstein and Lazar 2016). Also, the registry-nested design does allow for comparison with large groups of patients who underwent similar hip replacement surgery outside the study. Another limitation is that we do not collect radiographic outcomes for each participant.

The literature shows good survival rates up to 10 years for DM cups, ranging from 90.4% to 100% (Clave et al. 2016, Martz et al. 2017, Puch et al. 2017, Tarasevicius et al. 2017, Laurendon et al. 2018, Spaans et al. 2018, Cypres et al. 2019, Fessy et al. 2019, de l’Escalopier et al. 2020). Nevertheless, our population includes only patients aged 70 and older to minimize risk of revision for other indications such as loosening and wear. The study results may therefore promote additional research with a younger study population that is generally more active.

Important strengths of this study are that we will keep track of complications (serious adverse events) other than dislocations as well. In the long term, we shall be able to study survival of the implants as well as mortality in both study groups, as these remain available in the LROI. Finally, this trial not only evaluates effectiveness, but also the costs associated with both interventions. Such a trial-based economic evaluation is important to determine whether DM cups, which are typically more expensive, are worthwhile in a population undergoing primary THP.

### ^Ethics, registration, funding, and potential conflicts of interest^

This study (NL64819.100.18) is approved by the Medical research Ethics Committees United, the Netherlands, and will be conducted according to the principles of the Declaration of Helsinki (2013) and in accordance with the Medical Research Involving Human Subjects Act (WMO) and Good Clinical Practice guidelines.

The protocol of this trial is registered at clinicaltrials.gov (NCT04031820) and will be published. The main and secondary results of this study will be reported in international peer-reviewed journals.

This study is funded with a grant by the Van Rens Foundation, the Netherlands, identification number VRF2018-003.

No competing interests declared.
